# Supplementary material for: A rapid and robust leaf ablation method to visualize bundle sheath cells and chloroplasts in C3 and C4 grasses
Source: Plant Methods. 2023 Jul 6;19:69. doi: 10.1186/s13007-023-01041-x (PMC10324140; doi:10.1186/s13007-023-01041-x)
Supplement: Supplementary file 1 — Additional file 1: Movie showing rice leaf ablation. Rice leaf image with different vein orders before and after leaf ablation (top) and video showing the leaf ablation process (bottom). A drop of water was added onto a glass plate to prevent the dehydration while ablating the leaf. 1°: primary/mid vein; 2°: secondary/large lateral veins; 3°: tertiary/intermediate veins. Movie courtesy: Dr Satish Kumar Eeda. Additional file 2: Comparison of bundle cell width in paradermal versus transverse sections obtained from confocal laser scanning microscopy versus serial block-face scanning electron microscopy (SBF-SEM), respectively. (a) Transverse section of a rice leaf obtained from serial block-face scanning electron microscopy, representing the bundle sheath cells of a tertiary vein (3°). Bundle sheath cell width was measured at the mid-point of the medio-lateral axes as annotated with a red arrow. (b) Comparison of bundle sheath cell width measurements from paradermal and transverse sections, obtained from confocal imaging (rice data from Fig. 3c) and serial block-face scanning electron microscopy, respectively. BS: Bundle sheath cell; M: Mesophyll cell. Blue dot in the violin plots represent mean values. Statistical test: t-test. Additional file 3: Comparison of individual chloroplast areas obtained from confocal laser scanning microscopy versus serial block-face scanning electron microscopy (SBF-SEM). (a) Paradermal section of a rice leaf obtained from serial block-face scanning electron microscopy, representing the lateral bundle sheath cells of a tertiary vein (3°). Bundle sheath chloroplasts were pointed with red arrows. (b) Comparison of individual chloroplast areas from confocal (wild-type rice data from Fig. 4b) and two-dimensional serial block-face scanning electron microscopy imaging. BS: Bundle sheath cell; M: Mesophyll cell. Blue dot in the violin plots represent mean values. Statistical test: t-test. [file 13007_2023_1041_MOESM1_ESM.pptx]

## Slide 1
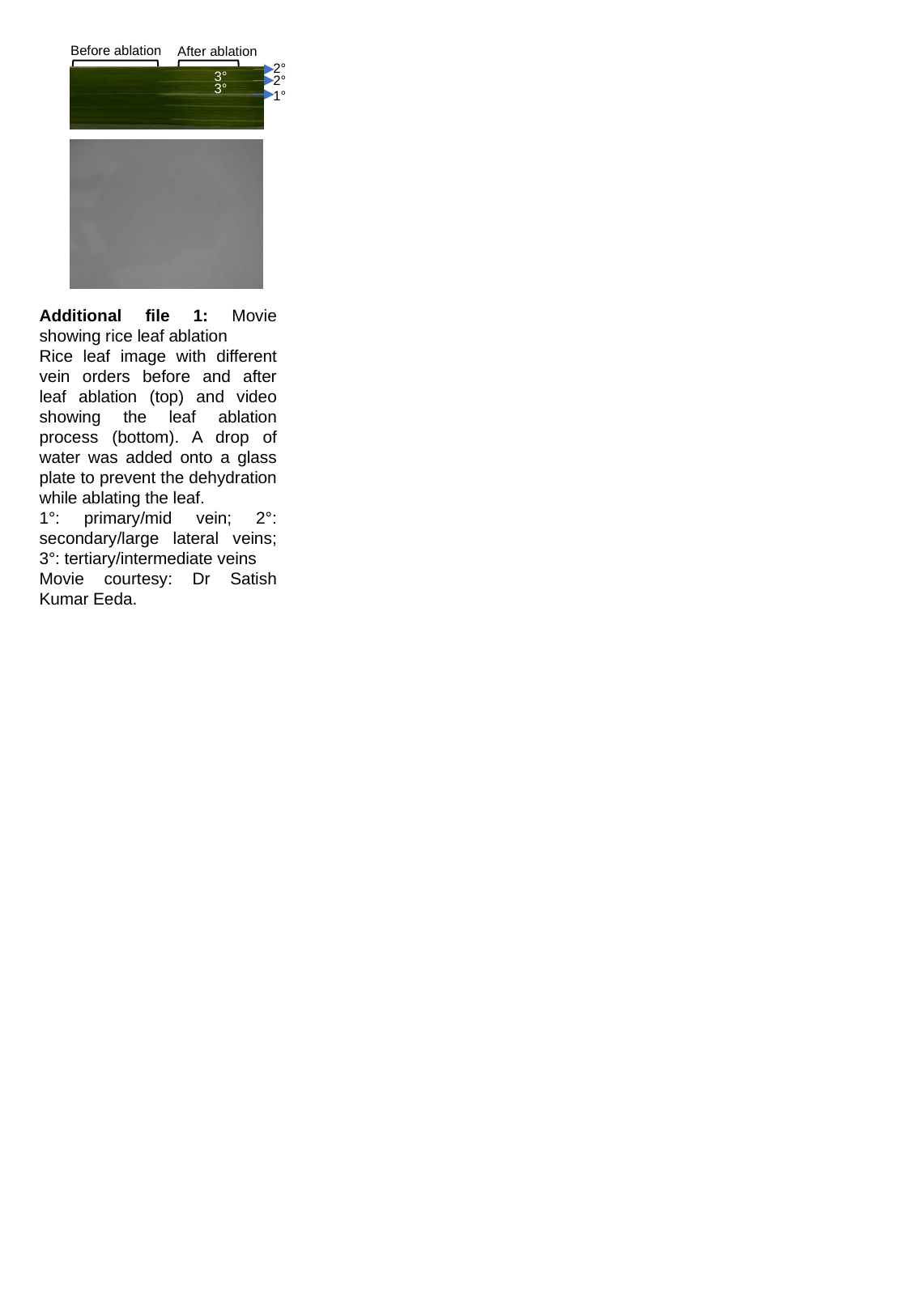

Before ablation
After ablation
2°
3°
2°
3°
1°
Additional file 1: Movie showing rice leaf ablation
Rice leaf image with different vein orders before and after leaf ablation (top) and video showing the leaf ablation process (bottom). A drop of water was added onto a glass plate to prevent the dehydration while ablating the leaf.
1°: primary/mid vein; 2°: secondary/large lateral veins; 3°: tertiary/intermediate veins
Movie courtesy: Dr Satish Kumar Eeda.

## Slide 2
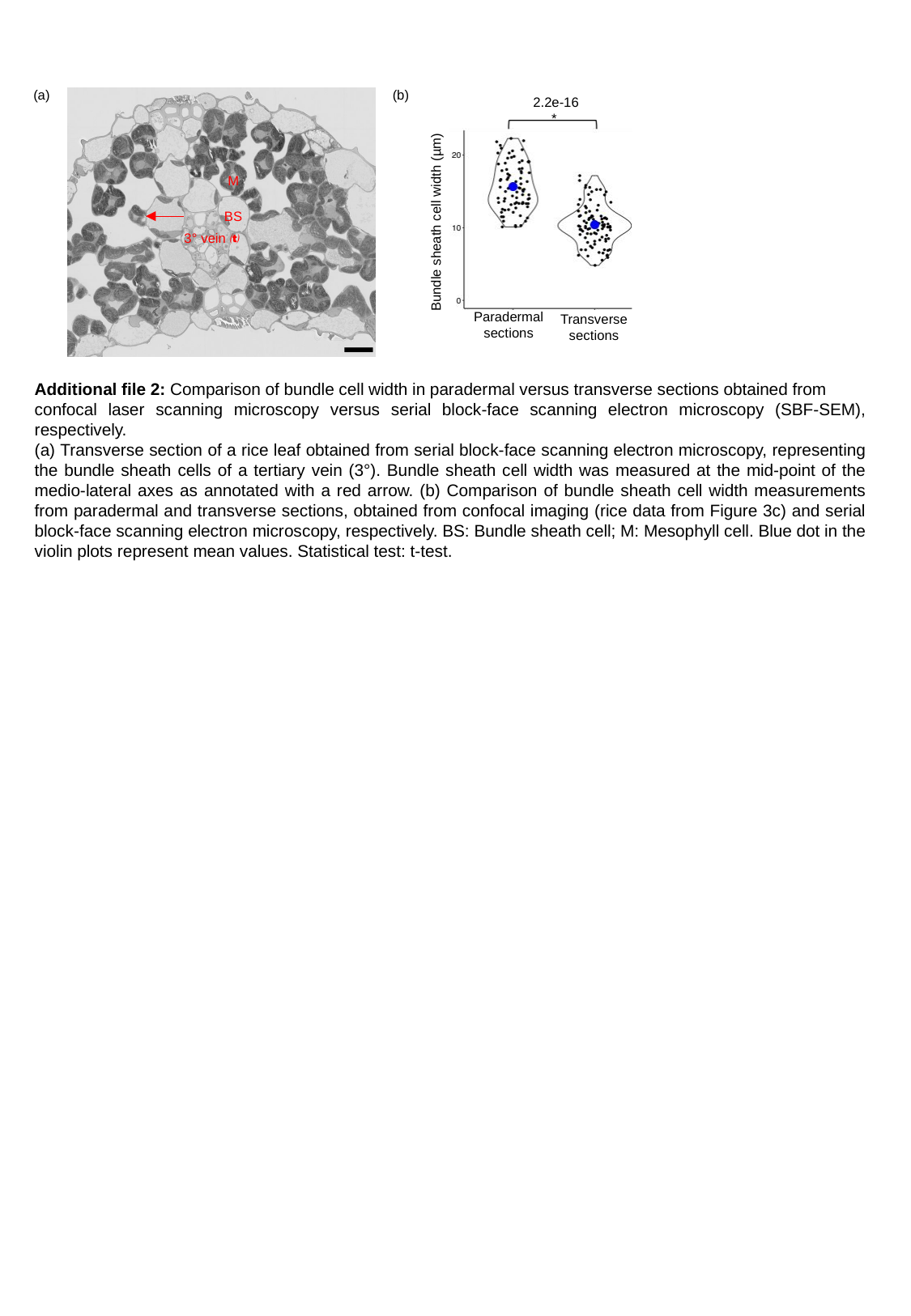

(b)
(a)
BS
M
3° vein 
 2.2e-16
*
Bundle sheath cell width (µm)
Paradermal
sections
Transverse
sections
Additional file 2: Comparison of bundle cell width in paradermal versus transverse sections obtained from
confocal laser scanning microscopy versus serial block-face scanning electron microscopy (SBF-SEM), respectively.
(a) Transverse section of a rice leaf obtained from serial block-face scanning electron microscopy, representing the bundle sheath cells of a tertiary vein (3°). Bundle sheath cell width was measured at the mid-point of the medio-lateral axes as annotated with a red arrow. (b) Comparison of bundle sheath cell width measurements from paradermal and transverse sections, obtained from confocal imaging (rice data from Figure 3c) and serial block-face scanning electron microscopy, respectively. BS: Bundle sheath cell; M: Mesophyll cell. Blue dot in the violin plots represent mean values. Statistical test: t-test.

## Slide 3
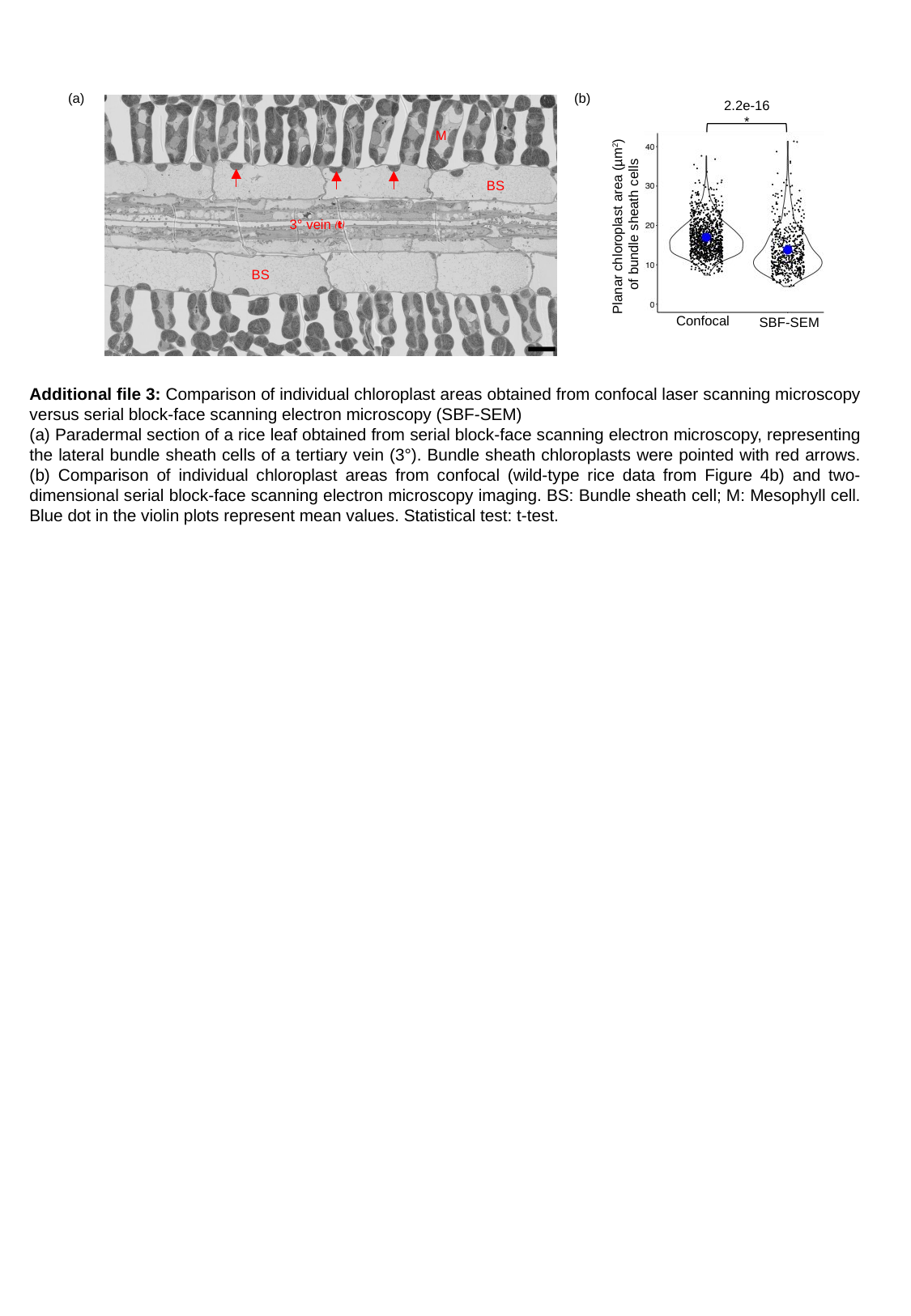

(a)
(b)
2.2e-16
*
Planar chloroplast area (µm2)
of bundle sheath cells
Confocal
SBF-SEM
M
BS
3° vein 
BS
Additional file 3: Comparison of individual chloroplast areas obtained from confocal laser scanning microscopy versus serial block-face scanning electron microscopy (SBF-SEM)
(a) Paradermal section of a rice leaf obtained from serial block-face scanning electron microscopy, representing the lateral bundle sheath cells of a tertiary vein (3°). Bundle sheath chloroplasts were pointed with red arrows. (b) Comparison of individual chloroplast areas from confocal (wild-type rice data from Figure 4b) and two-dimensional serial block-face scanning electron microscopy imaging. BS: Bundle sheath cell; M: Mesophyll cell. Blue dot in the violin plots represent mean values. Statistical test: t-test.
